# HMGB1: an important regulator of myeloid differentiation and acute myeloid leukemia as well as a promising therapeutic target

**DOI:** 10.1007/s00109-020-01998-5

**Published:** 2020-10-31

**Authors:** Lulu Liu, Jingjing Zhang, Xianning Zhang, Panpan Cheng, Lei Liu, Qian Huang, Haihui Liu, Saisai Ren, Peng Wei, Cuiling Wang, Cuiyun Dou, Lulu Chen, Xin Liu, Hao Zhang, Mingtai Chen

**Affiliations:** 1 Medical Research Center, Affiliated Hospital of Jining Medical University, Jining, 272029 Shandong Province China; 2 Department of Hematology, Affiliated Hospital of Jining Medical University, Jining, 272029 Shandong Province China; 3 Department of Radiation Oncology, Affiliated Hospital of Jining Medical University, Jining, 272029 Shandong Province China; 4 Department of Graduate School, Jining Medical University, Jining, 272000 Shandong Province China

**Keywords:** HMGB1, AML, Myeloid differentiation, TGFBI, Chidamide

## Abstract

**Abstract:**

High mobility group box 1 (HMGB1) is a non-histone nuclear protein which has been intensively studied in various physiological and pathological processes including leukemia. Here in this study, we further demonstrated that HMGB1 presents higher expression in the bone marrow mononuclear cells of acute myeloid leukemia (AML) patients compared with the normal controls and contributes to the AML pathogenesis and progression by inhibiting apoptosis, facilitating proliferation, and inducing myeloid differentiation blockade of AML cells. Mechanistic investigation revealed that transforming growth factor beta-induced (TGFBI) acts as a potential downstream target of HMGB1 and lentivirus-mediated knockdown of TGFBI expression impaired phorbol-12-myristate-13-acetate (PMA) and all-trans retinoic acid (ATRA)–induced myeloid differentiation of AML cell lines. On the other hand, chidamide, an orally histone deacetylase inhibitor, decreases HMGB1 expression significantly in AML cells with concomitant upregulation of TGFBI expression, and confers therapeutic effect on AML by inducing cell differentiation, apoptosis and inhibiting cell proliferation. In conclusion, our findings provide additional insights that HMGB1 is a promising therapeutic target of AML, and also present experimental evidence for the clinical application of chidamide as a novel agent in AML therapy by downregulating HMGB1 expression.

**Key messages:**

- HMGB1 induces cell proliferation and myeloid differentiation blockade and inhibits apoptosis of AML cells.
- TGFBI acts as a potential target of HMGB1.
- Chidamide, a selective HDAC inhibitor, confers promising therapeutic effect for AML via downregulating HMGB1 expression.

**Supplementary Information:**

The online version contains supplementary material available at 10.1007/s00109-020-01998-5.

## Introduction

The granulocytic and macrophage-like differentiation and maturation constitute the important part of myeloid differentiation which is a highly orchestrated process regulated by transcriptional factors (such as PU.1 and CCAAT/enhancer-binding protein(C/EBP)α) [1], cytokines (in particular granulocyte colony-stimulating factor (G-CSF), macrophage colony-stimulating factor (M-CSF), and interleukin-3 (IL-3)) [2], non-coding RNAs (miR-29a, miR-142-3p, and lnc-MC, etc.) [3, 4], and RNA-binding proteins [5]. Abnormal expression of these regulators can lead to acute myeloid leukemia (AML) which is a malignant hematopoietic neoplasm characterized by arrest of myeloid differentiation, rapid growth, and apoptotic repression of leukemic blasts arisen from the hematopoietic stem/progenitor cells (HSPCs) population within the bone marrow [6]. AML is the most common type of acute leukemia in adults. In the past three decades, great progress has been made in understanding AML pathogenesis and clinical treatment with novel agents and allogeneic stem cell transplantation [7–9]. However, the current AML therapeutic regimen only cures ~ 20% of the patients [10]. Thus, it is still of great importance and in urgent need to define the key regulator in myeloid differentiation and pathogenesis of AML and develop innovative agents and novel therapeutic strategies for AML treatment.

High mobility group box 1 (HMGB1) belongs to the HMG protein family and is a highly conserved non-histone nuclear protein that acts as a chromatin-binding factor for bending DNA and promoting access to transcriptional protein assemblies on specific DNA targets and contributes to the fine-tuning of transcription in response to rapid environmental changes [11, 12]. Additionally, increasing evidence demonstrate that HMGB1 can be released from cells under conditions of stress, such as injury, infection, and chemotherapy, and functions as damage-associated molecular pattern molecule (DAMP), eliciting immune and inflammatory response through interaction with the receptor for advanced glycation end products (RAGE) and some members of the Toll-like receptors (TLR2 and TLR4) [13]. As a multi-functional protein, HMGB1 has been widely investigated and considered an essential facilitator in diseases such as sepsis, collagen disease, atherosclerosis, cancers, arthritis, and acute lung injury and myocardial infarction [14]. There are also several studies indicating that HMGB1 plays an important role in leukemia pathogenesis and its upregulation is strongly associated with chemotherapy resistance by regulating cell autophagy [15, 16]. However, the function of HMGB1 in myeloid differentiation and AML remains to be elucidated, and also whether HMGB1 can be an effective therapeutic target in AML still needs more convincing experimental evidence.

Here in this study, we demonstrated that HMGB1 presents significantly high expression in AML patients and mediates the effect of facilitating proliferation and apoptotic repression on AML cells. Furthermore, HMGB1 is identified as a negative regulator of myeloid differentiation by influencing the expression of transforming growth factor beta-induced (TGFBI) which is demonstrated to promote phorbol-12-myristate-13-acetate (PMA) and all-trans retinoic acid (ATRA)–induced macrophage-like and granulocytic differentiation of AML cell lines. Chidamide, a histone deacetylase (HDAC) inhibitor, can significantly downregulate HMGB1 expression and confers therapeutic effect on AML by inducing cell differentiation, apoptosis, and inhibiting cell proliferation. These results further demonstrated the important role of HMGB1 in myeloid differentiation and the leukemogenesis of AML, and provide experimental evidence for the clinical application of chidamide in AML therapy by targeting HMGB1 expression.

## Materials and methods

### Bioinformatics analyses

Microarray data of AML expression profile (GSE79605), HMGB1 deletion (GSE18721), and chidamide treatment (GSE104280) were downloaded from GEO DataSets, and the differential genes were screened through bioinformatics analyses and act as clues for subsequent experimental validation.

### Human samples

The bone marrow samples of AML patients and iron deficiency anemia (IDA) normal controls were collected from the Hematology Department of the Affiliated Hospital of Jining Medical University. Informed consent to perform the biological studies was obtained from the individuals examined, and the related study was approved by the ethics committees of the hospitals and the Institutional Review Board of Jining Medical University. Mononuclear cell (MNC) fractions were isolated from the samples by Percoll density gradient [*d* = 1.077 g/ml] (Amersham Biotech, Germany), and CD34^+^ cells were enriched from MNCs through positive immunomagnetic selection (CD34 MultiSort kit, Miltenyi Biotec, Bergisch Gladbach, Germany). The characteristics of AML patients used in this study are presented in Table S1.

### Cell culture and differentiation induction

The following human cell lines were used in this study: THP-1 and HL-60 purchased from cell resource center of Shanghai Institutes for Biological Science, NB4 and 293TN purchased from cell resource center of Institute of Basic Medical Sciences, Chinese Academy of Medical Sciences. THP-1 and NB4 were cultured in PRMI 1640 medium (Hyclone); HL-60 was cultured in Iscove’s Modified Dubecco’s Medium (IMDM) (Hyclone); 293TN was cultured in Dulbecco’s Modified Eagle’s medium (DMEM) (Hyclone). All cultures were supplemented with 10% fetal bovine serum (FBS) (Hyclone), 100 U/ml penicillin, and 100 μg/ml streptomycin (Sigma-Aldrich, St. Louis, Mo, USA) at 37 °C in 5% CO_2_. The macrophage-like differentiation of THP-1 and HL-60 was induced with PMA (Sigma-Aldrich) at final concentration of 10 nM. The granulocytic differentiation of NB4 and HL-60 was induced with ATRA (Sigma-Aldrich) at final concentration of 2 μM. The CD34^+^ HSPC cells were cultured in IMDM with 30% FBS, 1% bovine serum albumin, 2 mM l-glutamine, 0.05 mM 2-mercaptoethanol, 50 U/ml penicillin, 50 μg/ml streptomycin, 50 ng/ml stem cell factor, and 20 ng/ml IL-3. The granulocytic and macrophage-like differentiation cultures of CD34^+^ HSPCs were performed as previously described [17]: 20 ng/ml G-CSF and 10 ng/ml IL-6 were added for granulocytic differentiation, and the cytokine cocktail of 50 ng/ml M-SCF, 1 ng/ml IL-6, and 100 ng/ml Flt-3L was used for macrophage-like differentiation. All of these cytokines were purchased from PeproTech (Rocky Hill, NJ, USA). Chidamide (Cat. No.: HY-13592) was purchased from MedChemExpress (MCE) and used to treat AML cells at final concentration of 2 μM.

### RNA Extraction and qRT-PCR analysis

Total RNA was extracted from cell samples using TRIzol reagent (Invitrogen) and quantified using the NanoDrop 2000 spectrophotometer (Thermo Scientific, Bremen, Germany). The first strand of cDNA was synthesized using Moloney murine leukemia virus (M-MLV) reverse transcriptase (Invitrogen) according to the manufacturer’s instructions. Oligo (dT) was used as the primer for reverse transcription of mRNA. GAPDH was used as the internal control. qRT-PCR was performed in a Bio-Rad CFX-96 System (Bio-Rad, Foster City, CA, USA) using the SYBR Premix (CWBio). The primers used for reverse transcription and qRT-PCR are listed in Table S2.

### Plasmid construction

The shRNA sequences for HMGB1 and TGFBI were synthesized, annealed, and inserted into pSIH1-H1-copGFP (System Biosciences). The oligonucleotides used for plasmid construction are listed in Table S3.

### Lentivirus production and cell infection

The recombination lentiviruses for knockdown were produced in 293TN cells using pSIH1-H1-copGFP-based constructs. Lentivirus packaging was performed using the pPACKH1 HIV Lentivector Packaging Kit (LV500A-1, System Biosciences, CA, USA) according to the manufacturer’s instructions. The culture supernatant containing the virus particles was directly used to infect the leukemia cells in 6-well plates with 5 μg/mL polybrene (Sigma-Aldrich).

### Cell proliferation assay

After lentiviruses infection, the cells were plated in 96-well plate at a density of 10,000 cells/well and incubated in 10% CCK-8 (Dojindo, Japan) diluted in normal culture medium at 37 °C for 1.5 h. Then, proliferation rates were determined at 0, 24, 48, 72, and 96 h by measuring the absorbance with a microplate reader set at 450 and 630 nm. All experiments were performed in triplicate.

### Cell apoptosis assay

The infected leukemia cells were starved in low FBS medium for 48 h and then collected, washed once with PBS, and re-suspended in the 1× binding buffer. Apoptotic cells were stained with APC-annexin V and 7-AAD (Biolegend) at room temperature for 20 min and immediately analyzed by flow cytometry.

### Flow cytometry analysis

The infected THP-1 and NB4 cells were induced towards macrophage-like and granulocytic differentiation, respectively, and harvested at 48 h of differentiation. The cells were rinsed twice with PBS, re-suspended in 100 μl PBS, and incubated with APC-conjugated anti-CD14 or anti-CD11b (eBioscience) at 4 °C for 30 min. Then, the cells were washed with 1 ml PBS, re-suspended in 300 μl PBS, and analyzed immediately using an AccuriC6 flow cytometer (BD, SD, USA).

### Western blot

The whole cell lysates were harvested and subjected to SDS-PAGE (10% separation gel) and transferred to a polyvinylidene difluoride (PVDF) membrane. Primary antibodies against the following proteins were used: HMGB1 (10829-1-AP; Proteintech), TGFBI (10188-1-AP; Proteintech), GAPDH (10494-1-AP; Proteintech), and actin (60008-1-Ig; Proteintech). Horseradish peroxidase–conjugated secondary antibodies were used (ZSGB-BIO). Signals were detected using an ECL (enhanced chemiluminescence) kit (Millipore).

### Statistical analysis

Student’s *t* test (two-tailed) was performed to analyze the data. Statistical significance was set at *P* < 0.05, as indicated by an asterisk (*P < 0.05; **P < 0.01).

## Results

### HMGB1 presents significantly high expression in AML patients

**Fig. 1:**
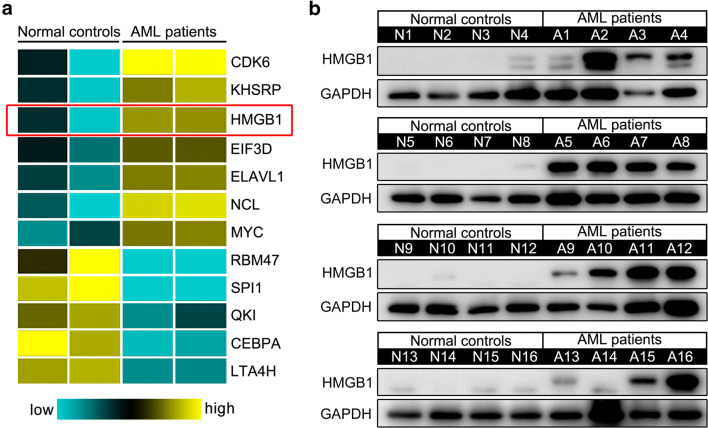
HMGB1 presents aberrant high expression in AML patients. **a** Partial gene expression profile in AML patients and normal controls was presented by analyzing the array data (GSE79605) annotated in GEO DataSets. **b** HMGB1 expression in 16 AML patients and 16 normal controls was analyzed by Western blot analysis. GAPDH was used as a loading control

To systematically identify key regulator involved in the leukemogenesis of AML, we first performed bioinformatics analysis using the expression profiling data of AML patients (GSE79605) annotated in the GEO DataSets. HMGB1 is defined as one of the significantly differential genes (Fig. 1a), and further Western blot analyses validated its higher expression in the bone marrow mononuclear cells (MNCs) of AML patients compared with the normal controls (Fig. 1b), which is in accordance with the findings previously reported [18] and implies important role of HMGB1 in the leukemogenesis of AML.

### HMGB1 regulates the proliferation and apoptosis of AML cells

**Fig. 2:**
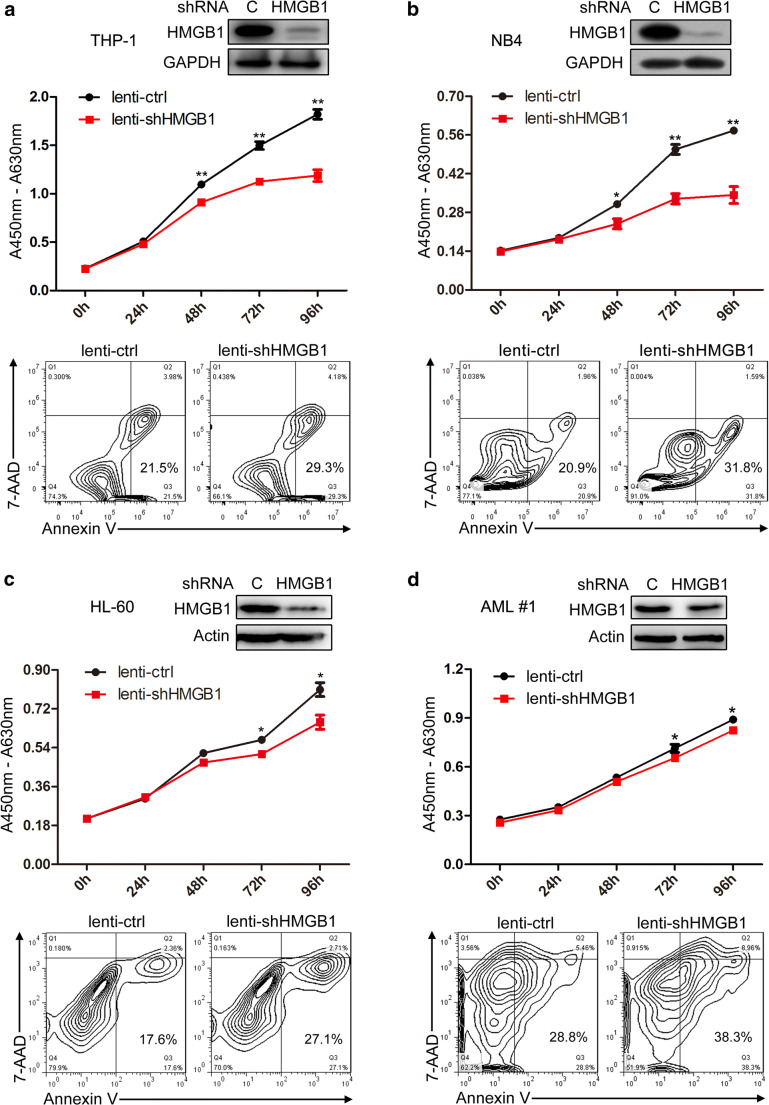
HMGB1 regulates the proliferation and apoptosis of AML cells. **a–d** THP-1, NB4, HL-60, and primary AML MNCs were infected with lenti-ctrl and lenti-shHMGB1, replaced with fresh medium 24 h later and cultured for another 48 h. Then, HMGB1 expression was detected by Western blot, cell proliferation was evaluated by CCK-8 assay, and cell apoptosis analyses were performed using double staining of annexin V and 7-AAD followed by flow cytometry assay. Knockdown of HMGB1 in **a** THP-1, **b** NB4, **c** HL-60, and **d** primary AML MNCs inhibits cell proliferation and promotes cell apoptosis. *P < 0.05 and **P < 0.01, Student’s *t* test

To investigate the role of HMGB1 in AML leukemogenesis, we make use of the recombined lentiviruses that express specific shRNA for HMGB1 (lenti-shHMGB1) to infect THP-1, NB4, HL-60, and primary AML MNCs. Western blot analyses revealed that lenti-shHMGB1 infection remarkably decreased HMGB1 expression which significantly inhibits proliferation and facilitate apoptosis of AML cells (Fig. 2a–d) as compared with the lenti-control (lenti-ctrl) infection. These results suggest that aberrant high expression of HMGB1 may participate in the progression of AML by contributing to cell proliferation and also repressing cell apoptosis.

### HMGB1 is identified as a negative regulator of myeloid differentiation

**Fig. 3:**
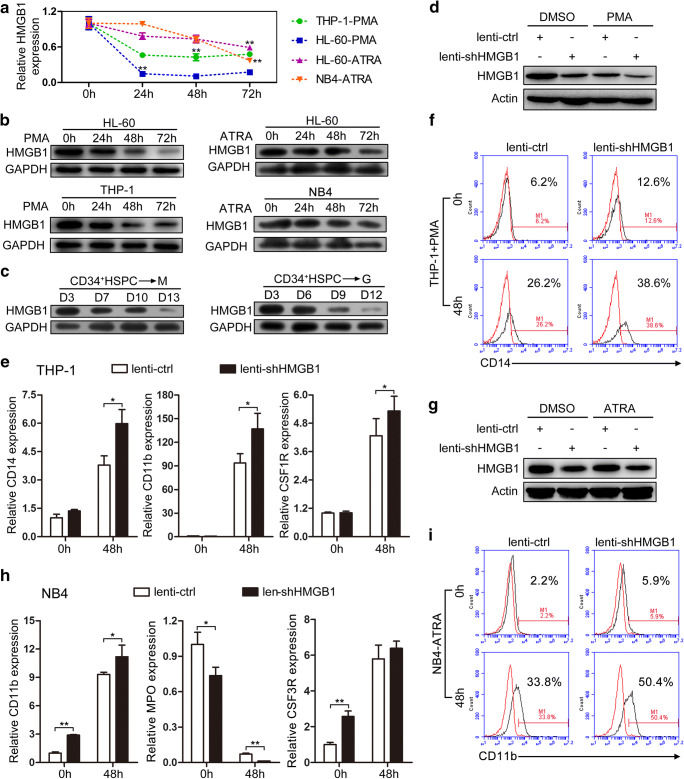
HMGB1 regulates myeloid differentiation. **a** qRT-PCR and **b** Western blot analyses of HMGB1 expression during PMA-induced macrophage-like differentiation of HL-60 and THP-1 cells and ATRA-induced granulocytic differentiation of HL-60 and NB4 cells. **c** Western blot analyses of HMGB1 expression during the in vitro macrophage-like and granulocytic differentiation of CD34^+^ HSPCs. **d** The expression of HMGB1 was analyzed by Western blot in THP-1 cells that were infected with lenti-shHMGB1 (or lenti-ctrl) followed by PMA induction for 48 h. **e** qRT-PCR detection of macrophage-like differentiation markers CD14, CD11b, and CSF1R in the infected and PMA-induced cells. Three independent experiments were performed, and data are means ± standard deviations. **f** CD14 expression was evaluated by cytometric analyses in infected and PMA-induced cells. Red and black curves show results for untreated cells and anti-CD14 antibody-stained cells, respectively. **g** The expression of HMGB1 was analyzed by Western blot in NB4 cells that were infected with lenti-shHMGB1 (or lenti-ctrl) followed by ATRA induction for 48 h. **h** qRT-PCR detection of granulocytic differentiation markers CD11b, MPO, and CSF3R in the infected and ATRA-induced cells. Three independent experiments were performed, and data are means ± standard deviations. **i** CD11b expression was evaluated by cytometric analyses in infected and ATRA-induced cells. Red and black curves show results for untreated cells and anti-CD11b antibody-stained cells, respectively. *P < 0.05 and **P < 0.01, Student’s *t* test.

AML development is often accompanied with myeloid differentiation blockade [19]. To evaluate whether aberrant expression of HMGB1 influences the myeloid differentiation process, we first detected the expression of HMGB1 using qRT-PCT and Western blot during the PMA-induced macrophage-like differentiation of HL-60 and THP-1 cells, and ATRA-induced granulocytic differentiation of HL-60 and NB4 cells. The results showed that HMGB1 presents gradually decreased expression during the PMA/ATRA-induced myeloid differentiation of AML cell lines at both mRNA and protein levels (Fig. 3a, b). Besides, we further validated the decreased HMGB1 protein expression during the in vitro cytokine-induced myeloid differentiation of CD34^+^ HSPCs derived from umbilical cord blood (Fig. 3c).

Next, we performed functional analysis to investigate whether HMGB1 participates in AML development by impeding myeloid differentiation through use of the PMA/ATRA-induced differentiation model of THP-1/NB4 leukemia cell lines. THP-1 cells were infected with lenti-shHMGB1 or lenti-ctrl, followed by PMA induction for 48 h. Western blot analyses revealed that lenti-shHMGB1 infection remarkably decreased HMGB1 expression which resulted in significant upregulation of mRNA level of the macrophage-like differentiation markers (CD14, CD11b, and CSF1R) (Fig. 3e) as compared with the lenti-ctrl infection. Flow cytometry analysis revealed increased CD14 expression upon lenti-shHMGB1 infection relative to lenti-ctrl infection (Fig. 3f). These results demonstrated that knockdown of HMGB1 expression facilitated the PMA-induced macrophage-like differentiation of THP-1 cells. On the other hand, NB4 cells were infected with lenti-shHMGB1 or lenti-ctrl, followed by ATRA induction for 48 h. Knockdown of HMGB1 expression was confirmed by Western blot analyses (Fig. 3g), which contributed to the upregulation of mRNA level of the granulocytic differentiation markers (CD11b, MPO, and CSF3R) as compared with the lenti-ctrl infection (Fig. 3h). The increased CD11b expression after HMGB1 knockdown, and ATRA induction in NB4 cells was also observed by flow cytometric assay (Fig. 3i). The results indicated that targeted inhibition of HMGB1 expression in NB4 cells promoted the ATRA-induced granulocytic differentiation.

Collectively, HMGB1 is identified as a negative regulator of myeloid differentiation and may participate in the AML development by inducing the differentiation blockade.

### TGFBI is verified as a target of HMGB1 and regulates myeloid differentiation

**Fig. 4:**
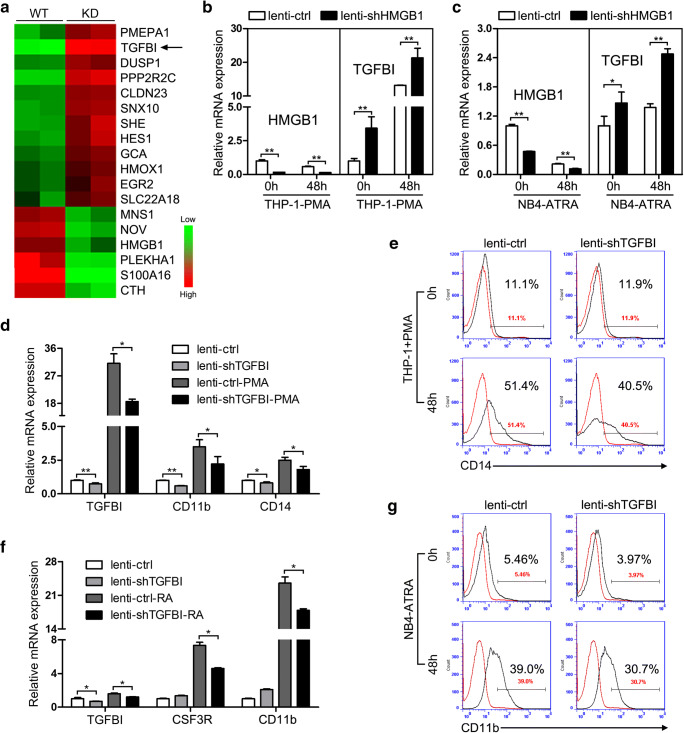
TGFBI is identified as a downstream target of HMGB1 and regulates myeloid differentiation. **a** Combined analysis of microarray data of HMGB1 deletion (GSE18721) and AML expression profile (GSE79605), partial differentially expressed genes were presented after HMGB1 deletion. **b** qRT-PCR analyses of HMGB1 and TGFBI expression in THP-1 cells that were infected with lenti-shHMGB1 (or lenti-ctrl) followed by PMA induction for 48 h. **c** qRT-PCR analyses of HMGB1 and TGFBI expression in NB4 cells that were infected with lenti-shHMGB1 (or lenti-ctrl) followed by ATRA induction for 48 h. **d** qRT-PCR detection of TGFBI expression and macrophage-like differentiation markers CD14 and CD11b in THP-1 cells that were infected with lenti-shTGFBI (or lenti-ctrl) followed by PMA induction for 48 h. **e** CD14 expression was evaluated by cytometric analyses in infected and PMA-induced cells. Red and black curves show results for untreated cells and anti-CD14 antibody-stained cells, respectively. **f** qRT-PCR detection of TGFBI expression and granulocytic differentiation markers CD11b and CSF3R in NB4 cells that were infected with lenti-shTGFBI (or lenti-ctrl) followed by ATRA induction for 48 h. **g** CD11b expression was evaluated by cytometric analyses in infected and ATRA-induced cells. Red and black curves show results for untreated cells and anti-CD11b antibody-stained cells, respectively. *P < 0.05 and **P < 0.01, Student’s *t* test.

To reveal the molecular mechanism of HMGB1 regulating myeloid differentiation and AML leukemogenesis, we first performed bioinformatics analyses of the expression profile after HMGB1 deletion (GSE18721). Combined with the expression profile in AML patients (GSE79605), several differentially expressed genes after HMGB1 deletion were selected, and finally, we focused on TGFBI which is a transforming growth factor beta (TGF-β)–induced protein and presents significantly high expression after HMGB1 deletion (Fig. 4a). Experimental validation indicated that knockdown of HMGB1 expression in THP-1 and NB4 cells resulted in obvious increased expression of TGFBI in both before and after PMA/ATRA-induced differentiation (Fig. 4b, c). Remarkable upregulation of TGFBI upon PMA/ATRA induction was also observed (Fig. 4b, c), implicating its potential role in myeloid differentiation.

To evaluate the effect of TGFBI on the differentiation, we use the recombined lentiviruses that express specific shRNA for TGFBI (lenti-shTGFBI) to infect THP-1 and NB4 cells followed by PMA and ATRA induction, respectively, for 48 h. As expected, lenti-shTGFBI infection of THP-1 cells resulted in significant downregulation of TGFBI expression and impaired the PMA-induced macrophage-like differentiation as revealed by the decreased mRNA expression of differentiation markers (CD11b and CD14) compared with the lenti-ctrl infection (Fig. 4d). Decreased CD14 expression in lenti-shTGFBI-infected and PMA-induced THP-1 cells was also observed evaluated by flow cytometric assay (Fig. 4e). In the meanwhile, lentivirus-mediated knockdown of TGFBI in NB4 cells also presented similar results that decreased expression of TGFBI attenuated ATRA-induced granulocytic differentiation characterized by the downregulation of granulocytic differentiation markers detected by qRT-PCR and flow cytometry (Fig. 4f, g).

Taken together, these results demonstrated that TGFBI could act as a potential downstream target of HMGB1 and positively regulates PMA/ATRA-induced myeloid differentiation of AML cell lines.

### Chidamide downregulates HMGB1 expression and confers therapeutic effect in AML

**Fig. 5:**
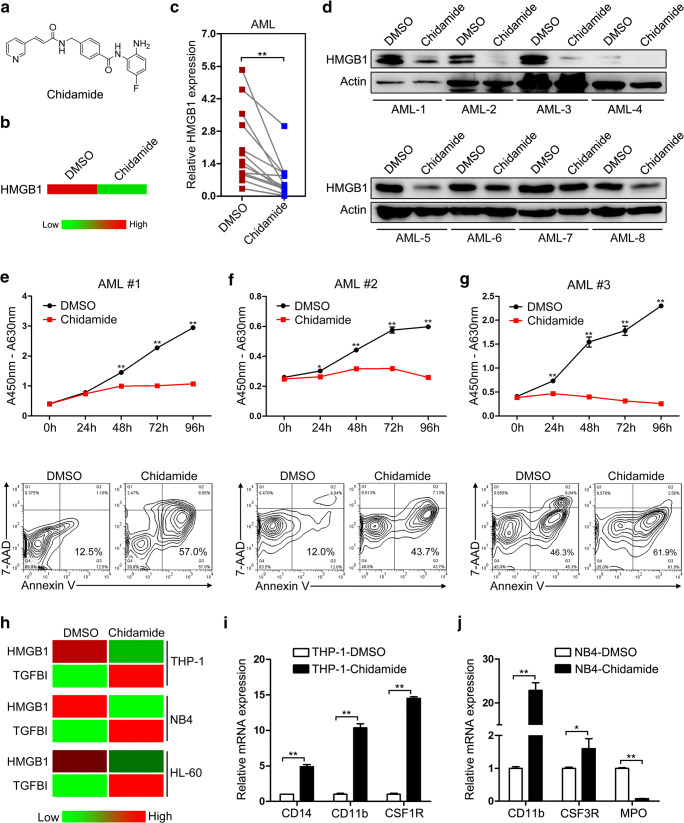
Chidamide downregulates HMGB1 expression and confers therapeutic effect in AML. **a** Chemical structure of chidamide. **b** HMGB1 expression after chidamide treatment indicated by the RNA-Seq data (GSE104280). **c** qRT-PCR and **d** Western blot analyses of HMGB1 expression in chidamide-treated AML MNCs. The actin band in the top panel of Fig. 5d was the same as that in Fig. S1B, as the data were collected in one experiment using the same samples. **e**–**g** The bone marrow MNCs were isolated from three independent AML patients and treated with chidamide for indicated times. Then, cell proliferation was evaluated by CCK-8 assay and cell apoptosis analyses were performed using double staining of annexin V and 7-AAD followed by flow cytometry assay. **h** qRT-PCR detection of HMGB1 and TGFBI expression in chidamide-treated THP-1, NB4, and HL-60 cells. **i** qRT-PCR analyses of macrophage-like differentiation markers CD14, CD11b, and CSF1R in chidamide-treated THP-1 cells. **j** qRT-PCR analyses of granulocytic differentiation markers CD11b, CSF3R, and MPO in chidamide-treated THP-1 cells. *P < 0.05 and **P < 0.01, Student’s *t* test

Our results indicate that targeted inhibition of HMGB1 expression in AML can suppress cell proliferation, induce cell apoptosis, and overcome differentiation blockade, implicating HMGB1 as a promising therapeutic target in AML. Unexpectedly, through bioinformatics analyses, we found that chidamide, a novel orally subtype-selective HDAC inhibitor, whose chemical structure is presented in Fig. 5a, exerts significant suppressive effect on HMGB1 expression (Fig. 5b). To test whether chidamide regulates HMGB1 expression in AML cells, primary MNCs derived from the bone marrow of AML patients were separated and treated with chidamide for 48 h. Chidamide treatment led to remarkable downregulation of HMGB1 expression at both mRNA and protein levels (Fig. 5c, d), with concomitantly increased TGFBI expression in the partial AML samples (Fig. S1). To investigate the therapeutic effect of chidamide on AML, cell proliferation and apoptosis assays were performed after chidamide handling. In agreement with HMGB1 expression, chidamide presented striking inhibitory effect on the proliferation of primary AML cells (Fig. 5e–g, top panel) and remarkably facilitated cell apoptosis (Fig. 5e–g, bottom panel). On the other hand, chidamide treatment also led to significant downregulation of HMGB1 expression accompanied with upregulation of TGFBI expression in THP-1, NB4, and HL-60 cells (Fig. 5h). In the meanwhile, apparent proliferation repression of THP-1 and NB4 cells was also observed in the culture medium with chidamide (Fig. S2); macrophage-like and granulocytic differentiation markers also presented significant upregulation with chidamide treatment alone (Fig. 5i, j). Altogether, these results indicated that chidamide can contribute to the striking downregulation of HMGB1 expression and confers promising therapeutic effect on AML by regulating the proliferation, apoptosis, and differentiation of AML cells.

## Discussion

AML is a heterogeneous disorder of aggressive hematopoietic disease characterized by malignant proliferation and apoptotic repression of clonal neoplastic cells and also differentiation arrest of myeloid blasts [20, 21]. HMGB1, as a critical anti-apoptotic and pro-autophagic protein, has been reported to be overexpressed and released following chemotherapy and radiation therapy in many cancers including leukemia cells [22]. High expression of HMGB1 is associated with the progression of lymphocytic leukemia and chronic myeloid leukemia [23]. The extracellular and endogenous HMGB1 could also activate autophagy through PI3K-MEK-ERK and PI3K/Akt/mTORC1 pathways and mediate the drug resistance to chemotherapy in leukemia cells [16, 24]. Here in this study, our results demonstrated that HMGB1 is involved in the development and progression of AML not only by inhibiting cell apoptosis and promoting cell proliferation but also through acting as a negative regulator of myeloid differentiation, which provides more experimental evidence for conferring HMGB1 as a therapeutic target for AML.

As a multi-functional protein, HMGB1 is widely expressed and has been intensively studied in various physiological and pathological processes [14]. HMGB1 is first identified as a chromatin-binding chaperon protein containing two DNA binding boxes (N-terminal A and central B) along with an acidic C-terminal tail, which binds to DNA and modulates the architecture of the chromosome, thereby regulating gene transcription, DNA recombination, and repair [25]. Increasing evidence indicate that extracellular HMGB1 released from the cell could act as an inflammatory signal to elicit inflammation and immune response and participate in the late-stage pathogenesis of sepsis [26]. HMGB1 is also implicated as a potential RNA-binding protein regulating RNA metabolism at the post-transcriptional level [27], although its direct downstream targets have not yet been identified and reported. In this study, we found that TGFBI was negatively regulated by HMGB1 during the PMA/ATRA-induced myeloid differentiation of AML cell lines. As to the detailed molecular mechanism of how HMGB1 regulates TGFBI expression transcriptionally or post-transcriptionally during myeloid differentiation, it was not fully elucidated in this study. Our previous work found that TGFBI, highly expressed in the bone marrow mesenchymal stem cells, is identified as a direct target of miR-199a-5p and negatively regulates adipogenic differentiation [28]. MiR-199a-5p is also reported to inhibit monocyte/macrophage differentiation by targeting ACVR1B-mediated activation of the TGF-β signaling pathway [29]. As a TGF-β-induced protein, we demonstrated that TGFBI acts as a positive regulator and facilitates PMA/ATRA-induced myeloid differentiation of AML cell lines. However, obvious influence on cell proliferation and apoptosis was not observed after TGFBI interference (data not shown), which may be due to the low-abundance expression of TGFBI in AML cells.

HDACs are enzymes involved in remodeling of chromatin by deacetylating the lysine residues and play important roles in cancer development by regulating the expression and activity of numerous proteins involved in cancer initiation and progression [30, 31]. Aberrant activation or overexpression of HDACs has been reported in various cancers including leukemia [32, 33], and HDAC inhibitors represent a new class of targeting agents that have been developed and still in different stages of clinical trials for the treatment of both hematologic and solid malignancies [34, 35]. Chidamide is a novel and orally available benzamide-type HDAC inhibitor that selectively inhibits the activity of HDAC1, 2, 3, and 10 [36, 37]. It is developed by Chipscreen Biosciences and has gained approval from China Food and Drug Administration in 2015 for the treatment of relapsed/refractory peripheral T cell lymphoma [38, 39]. Moreover, chidamide is also being investigated in preclinical and clinical studies through use alone or in combination with other chemotherapeutic agents for other types of hematological cancers including non-Hodgkin’s lymphoma, diffuse large B cell lymphoma, and AML [40–42]. Here our results demonstrated that chidamide alone presents promising therapeutic effect on AML by strikingly inhibiting proliferation, promoting apoptosis, and inducing differentiation of AML cells. Active mechanisms of chidamide as an anti-cancer agent are manifold and involve various biological processes such as cell cycle arrest, apoptosis, differentiation, and amplification of immune cell-mediated cytotoxicity targeting tumor cells [36, 43]. Chidamide has been reported to exert its function mainly by upregulating the post-translational acetylation status of histones and non-histone proteins, which increases expression of various genes associated with the growth and survival of tumor cells, decreases the expression of Bcl-2 family and other pro-survival proteins, and upregulates some molecular markers recognized by immune cells and other chemotherapy drugs [44–46]. In this study, HMGB1 presents significant decreased expression upon chidamide treatment in AML cells and acts as a potential target of chidamide mediating the therapeutic effect on AML. Although the molecular detail about how chidamide decreases HMGB1 expression in AML cells remains to be elucidated, our results provide experimental clue for targeting HMGB1 expression using chidamide as a novel strategy in AML therapy.

In summary, our findings provide additional insights that HMGB1 contributes to the AML pathogenesis and progression by inhibiting apoptosis, facilitating proliferation, and inducing differentiation arrest of AML cells, acting as a promising therapeutic target in AML, and further demonstrating the therapeutic effect of chidamide on AML by reversing the biological processes correlated with the pathogenesis and progression of AML via downregulating HMGB1 expression.

## Supplementary Information

ESM 1 (DOC 433 kb)

## Data Availability

All the data and material in the manuscript are available and comply with the field standards.
