# Supplementary material for: HMGB1: an important regulator of myeloid differentiation and acute myeloid leukemia as well as a promising therapeutic target
Source: J Mol Med (Berl). 2020 Oct 31;99(1):107–18. doi: 10.1007/s00109-020-01998-5 (PMC7782413; doi:10.1007/s00109-020-01998-5)
Supplement: Supplementary file 1 — (DOC 433 kb) [file 109_2020_1998_MOESM1_ESM.doc]

**Supplemental Material**

**HMGB1: an important regulator of myeloid differentiation and acute myeloid leukemia as well as a promising therapeutic target**

Lulu Liu^1,5^, Jingjing Zhang ^2,5^, Xianning Zhang ^1,5^, Panpan Cheng ^2,5^, Lei Liu ^2^, Qian Huang ^2^, Haihui Liu ^2^, Saisai Ren ^2^, Peng Wei ^3^, Cuiling Wang ^2^, Cuiyun Dou ^2^, Lulu Chen ^4^, Xin Liu ^4^, Hao Zhang ^2^, Mingtai Chen ^1, *^

**
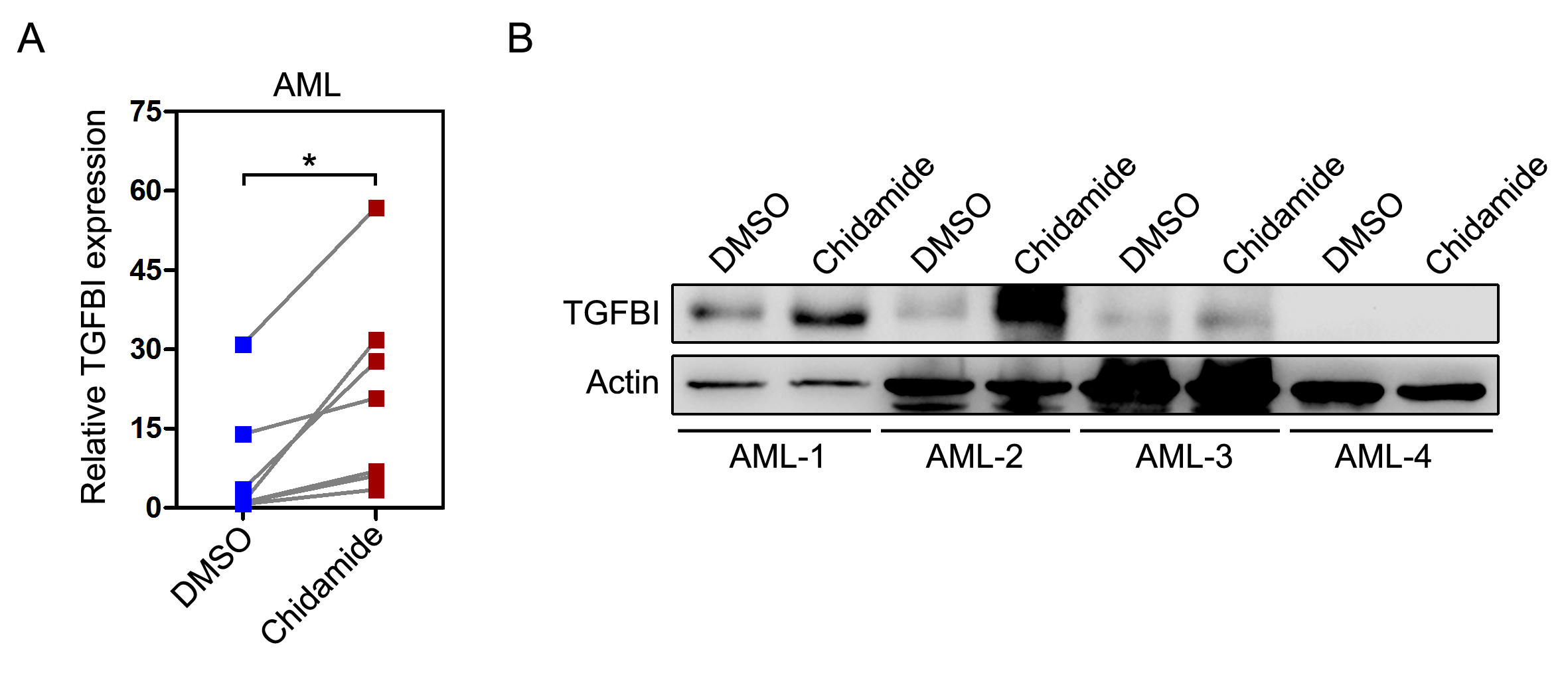
**

Figure S1. Chidamide up-regulates TGFBI expression. (A-B) qRT-PCR (A) and western blot (B) analyses of TGFBI expression in Chidamide-treated primary AML MNCs. The actin band was the same as that in Fig.5D (top panel), as the data were collected in one experiment using the same samples.

**
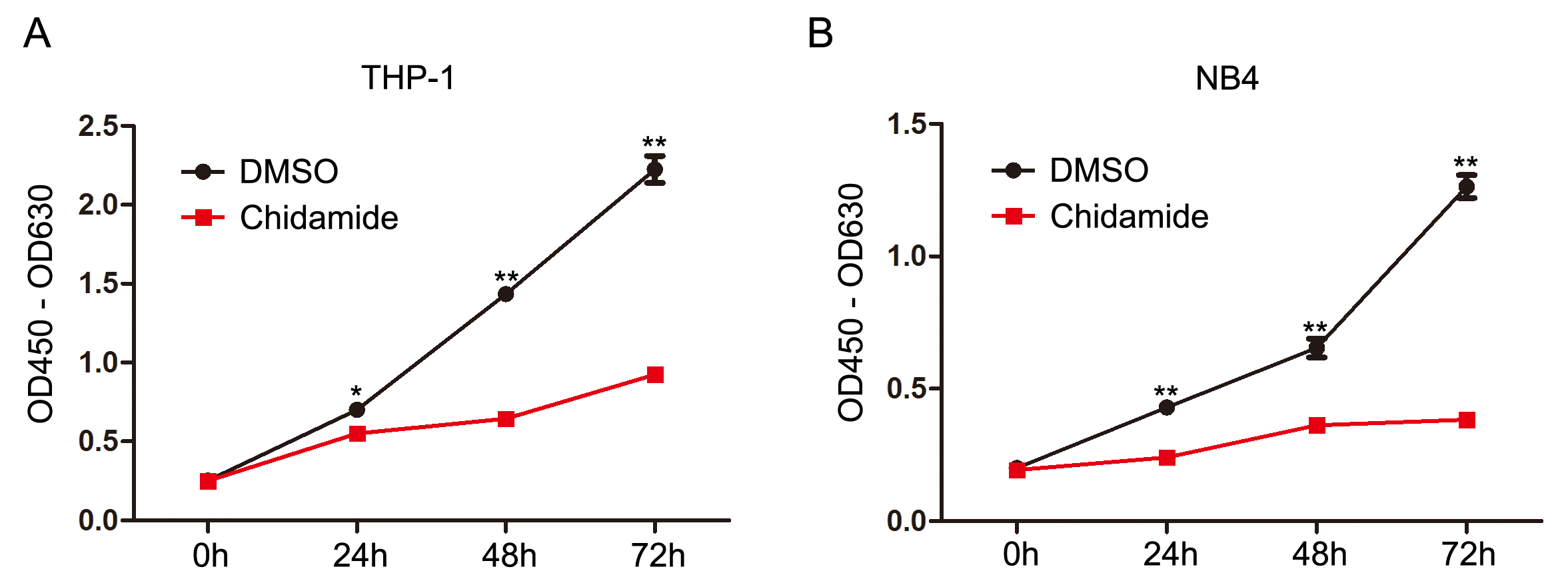
**

Figure S2. Chidamide inhibits the proliferation of THP-1 and NB4 cell lines. (A-B) THP-1 (A) and NB4 (B) cells were treated by Chidamide and the proliferation was evaluated by CCK-8 assay.

**Table S1. Characteristics of AML patients used in this study.**

| No. | Age (years) | Sex | WBC count (10^9/L) | blast% in BM | FAB | Fusion and mutation status | Cytogenetics |
| --- | --- | --- | --- | --- | --- | --- | --- |
| 1 | 52 | M | 3.33 | 92 | M3 | PML-RARα | 46,XY,t(15；17)(q22;q21) |
| 2 | 55 | F | 3.4 | 28 | M4 | - | Normal |
| 3 | 51 | M | 56.33 | 89 | M3 | - | Normal |
| 4 | 32 | M | 3.11 | 71.8 | M4 | WT1(+) | Normal |
| 5 | 26 | F | 342.23 | 93 | M3 | - | Normal |
| 6 | 42 | M | 3.77 | 35 | M2 | NPM1(+) | Complex |
| 7 | 76 | M | 71.29 | 69 | M2 | - | Normal |
| 8 | 33 | M | 36.7 | 47.5 | M4eo | CBFβ-MYH11 | 46,xy,inv(16)(p13,q22) |
| 9 | 49 | F | 3.8 | 80 | M3 | PML-RARα WT1(+) | 46，XX，t(15,17)(q22,q21) |
| 10 | 15 | M | 12.95 | 61.5 | - | TLS-ERG | 46,XY,t(16,21)(p11,q22) |
| 11 | 62 | F | 7.22 | 83 | M2 | NRAS(+) CEBPA(+) | 43～45,XX,del(7)(p15),-21[cp20] |
| 12 | 33 | F | 53.09 | 92 | M5 | - | Normal |
| 13 | 72 | M | 121.98 | 70.5 | M4b | - | 42,XY,add (1)(p36),der(7；15)（q10；q10）, add (8)（q24） |
| 14 | 68 | M | 22.3 | 37.5 | M4 | - | Normal |
| 15 | 19 | M | 26.64 | 31 | M5 | - | 46,XY,t(2;7)(p12;p15) |
| 16 | 61 | M | 0.64 | 73 | M2 | AML-ETO | 45, X,-Y，t（8；21）（q22；q22） |
| 17 | 44 | F | 18.81 | 49 | - | IDH2 (+) NPM1 (+) | Normal |
| 18 | 43 | F | 17.33 | 79.5 | M4 | CBFβ-MYH11 | 46, X,del（X)(q21), inv（16）（p13q22） |
| 19 | 68 | F | 105.97 | 67.5 | - | RUNX1 (+) ASXL1 (+) NRAS(+) SETBP1 (+) | Normal |
| 20 | 62 | M | 61.64 | 90.5 | M5 | TLS-ERG | 46,XY, del（6)(q21） |
| 21 | 68 | F | 128.12 | 82 | - | FLT3-ITD SF3B1 (+) AML1 (+) | Normal |
| 22 | 18 | M | 196.66 | 97 | M5 | MLL-AF6 | 46,XY, i(20)(q10） |
| 23 | 87 | F | 34.34 | 87 | M5 | - | Normal |
| 24 | 55 | M | 7.9 | 51 | M2 | AML-ETO KIT (+) | 46,XY,t(8;21)(q22;q22) |
| 25 | 45 | F | 1.35 | 53 | - | CEBPA (+) | Normal |

| 26 | 67 | M | 2.47 | 40 | - | TET2 (+) IDH1 (+) | Normal |
| --- | --- | --- | --- | --- | --- | --- | --- |
| 27 | 57 | F | 156.4 | 89 | M5 | FLT3-TKD NPM1 (+) DNMT3A (+) IDH2 (+) | Normal |
| 28 | 31 | F | 156.94 | 95 | M3 | PML-RARα FLT3-ITD | 46,XX,t(15,17)(q22,q21) |
| 29 | 68 | M | 26.76 | 59 | M5 | TET2 (+) NRAS(+) | Normal |
| 30 | 30 | F | 17.12 | 43 | M4 | CEBPA(+) | Normal |
| 31 | 54 | M | 3.95 | 61 | M5 | U2AF1 (+) ETV6 (+) | Normal |
| 32 | 25 | F | 24.06 | 66 | M5 | NPM1 (+) DNMT3A (+) CEBPA(+) IDH1 (+) | Normal |

**Table S2.** **Primers used for reverse transcription and qRT-PCR.**

| Name | Primers |
| --- | --- |
| HMGB1-F | TATGGCAAAAGCGGACAAGG |
| HMGB1-R | GCTTCGCAACATCACCAATGGA |
| GAPDH-F | GGAGCGAGATCCCTCCAAAAT |
| GAPDH-R | GGCTGTTGTCATACTTCTCATGG |
| Actin-F | CTGGCACCACACCTTCTACA |
| Actin-R | AGCACAGCCTGGATAGCAAC |
| CD11b-F | GGGCTGGTGGAGTCTTTCTAT |
| CD11b-R | TTCTGCCTGAACATCGCTA |
| CD14-F | CGCTCCGAGATGCATGTG |
| CD14-R | TTGGCTGGCAGTCCTTTAGG |
| CSF1R-F | CCTGAAGGTGGCTGTGAAGATG |
| CSF1R-R | GCTCCCAGAAGGTTGACGATG |
| MPO-F | GCACGCCCAACAACATCGAC |
| MPO-R | ATGCTGAACACACCCTCGTT |
| CSF3R-F | TCAAGTTGGTGCTATGGCAAGG |
| CSF3R-R | GCTCCCAGTCTCCACAGAATC |
| TGFBI-F | CTTCGCCCCTAGCAACGAG |
| TGFBI-R | TGAGGGTCATGCCGTGTTTC |
| oligodT | TTTTTTTTTTTTTTTTTT |

**Table S3** Primers and oligonucleotides used for plasmid construction.

| Name | Primers and oligonucleotides |
| --- | --- |
| shcon-F | TGAACTCAAGACCGATATTATTCAAGAGATAATATCGGTCTTGAGTTCTTTTTTC |
| shcon-R | TCGAGAAAAAAGAACTCAAGACCGATATTATCTCTTGAATAATATCGGTCTTGAGTTCA |
| HMGB1-shRNA-F | GATCCGGAGAGATGTGGAATAACATTCAAGAGATGTTATTCCACATCTCTCCTTTTTG |
| HMGB1-shRNA-R | AATTCAAAAAGGAGAGATGTGGAATAACATCTCTTGAATGTTATTCCACATCTCTCCG |
| TGFBI-shRNA-F | GATCCGCACTAATAGGAAGTACTTTTCAAGAGAAAGTACTTCCTATTAGTGCTTTTTG |
| TGFBI-shRNA-R | AATTCAAAAAGCACTAATAGGAAGTACTTTCTCTTGAAAAGTACTTCCTATTAGTGCG |
